# Polyethylene Glycol 35 (PEG35) Protects against Inflammation in Experimental Acute Necrotizing Pancreatitis and Associated Lung Injury

**DOI:** 10.3390/ijms21030917

**Published:** 2020-01-30

**Authors:** Ana Ferrero-Andrés, Arnau Panisello-Roselló, Anna Serafín, Joan Roselló-Catafau, Emma Folch-Puy

**Affiliations:** 1 Experimental Pathology Department, Institut d’Investigacions Biomèdiques de Barcelona-Consejo Superior de Investigaciones Científicas (IIBB-CSIC), Barcelona, 08036 Catalonia, Spain; anna.fan0311@gmail.com (A.F.-A.); arnau.panisello@iibb.csic.es (A.P.-R.); 2 PCB Animal Facility-Parc Científic de Barcelona, Barcelona, 08028 Catalonia, Spain; amserafin@pcb.ub.es; 3 Experimental Pathology Department, Institut d’Investigacions Biomèdiques de Barcelona-Consejo Superior de Investigaciones Científicas (IIBB-CSIC), Centro de Investigación Biomédica en Red de Enfermedades Hepáticas y Digestivas (CIBEREHD), Institut d’Investigacions Biomèdiques August Pi i Sunyer (IDIBAPS), Barcelona, 08036 Catalonia, Spain; joan.rosello@iibb.csic.es

**Keywords:** pancreatitis, inflammation, pulmonary injury, polyethylene glycols, cytokines

## Abstract

Acute pancreatitis is an inflammatory disorder of the pancreas. Its presentation ranges from self-limiting disease to acute necrotizing pancreatitis (ANP) with multiorgan failure and a high mortality. Polyethylene glycols (PEGs) are non-immunogenic, non-toxic, and water-soluble chemicals composed of repeating units of ethylene glycol. The present article explores the effect of PEG35 administration on reducing the severity of ANP and associated lung injury. ANP was induced by injection of 5% sodium taurocholate into the biliopancreatic duct. PEG35 was administered intravenously either prophylactically or therapeutically. Three hours after ANP induction, pancreas and lung tissue samples and blood were collected and ANP severity was assessed. To evaluate the inflammatory response, gene expression of pro-inflammatory cytokines and chemokine and the changes in the presence of myeloperoxidase and adhesion molecule levels were determined in both the pancreas and the lung. To evaluate cell death, lactate dehydrogenase (LDH) activity and apoptotic cleaved caspase-3 localization were determined in plasma and in both the pancreatic and lung tissue respectively. ANP-associated local and systemic inflammatory processes were reduced when PEG35 was administered prophylactically. PEG35 pre-treatment also protected against acute pancreatitis-associated cell death. Notably, the therapeutic administration of PEG35 significantly decreased associated lung injury, even when the pancreatic lesion was equivalent to that in the untreated ANP-induced group. Our results support a protective role of PEG35 against the ANP-associated inflammatory process and identify PEG35 as a promising tool for the treatment of the potentially lethal complications of the disease.

## 1. Introduction

Acute pancreatitis, an inflammatory disorder of the pancreas, is the main cause of hospitalizations related to gastrointestinal diseases and the fifth most common cause of death in hospitals [1]. This disease entity can be divided into two morphological subtypes [2]: interstitial edematous pancreatitis, and necrotizing pancreatitis. Interstitial edematous acute pancreatitis represents 80–90% of cases and the clinical symptoms usually resolve within the first week. However, up to 20% of patients develop acute necrotizing pancreatitis (ANP), the more severe form, which is associated with high rates of morbidity and mortality; lung failure is the main contributing factor to early death within the first week after admission [3]. At present, there is no specific medical treatment for ANP: the management of the disease is mainly supportive and targeted to prevent and treat systemic complications. 

Polyethylene glycols (PEGs) are non-immunogenic, non-toxic, and water-soluble polymers composed of repeating units of ethylene glycol [4]. One of their most positive characteristics is their low toxicity regardless of the route of administration [5]. In fact, PEGs are currently the only water-soluble polymers that are widely accepted and approved by the Food and Drug Administration for use in food, cosmetics, and pharmaceuticals [6]. In clinical settings, PEGs are used as additives to organ preservation solutions to attenuate the damage associated with cold ischemia-reperfusion of kidney, pancreas, and liver transplantation [7]. 

In recent years, several experimental studies have focused on the protective effects of PEGs. In lung endothelial cells, treatment with 15–20 kDa molecular weight PEG (PEG 15–20) was found to enhance cell function by activating endothelial cell-barrier signal transduction pathways and by contributing to cytoskeleton reorganization [8]. In addition, pre-treatment with PEG 15–20 in cultured ventricular myocytes subjected to hypoxia-reoxygenation reduced oxidative stress and apoptosis and increased cell survival [9]. The protective effects of PEGs have also been reported in in vivo models, although reports of its use in the context of inflammatory processes are scarce. In a murine model of lethal gut-derived sepsis, the therapeutic administration of low molecular weight PEG provided protection against bacterial infections and reduced mortality [10]. Likewise, the prophylactic oral administration of 4-kDa molecular weight PEG in experimental colitis strengthened the epithelial barrier and reduced inflammation of the colon [11]. Another study found that coating the peritoneal surfaces of the rat with PEG was a highly effective measure to limit the number of leukocytes in a model of post-traumatic inflammation [12]. Lastly, in colon carcinogenesis, animals receiving a diet with 8-kDa molecular weight PEG presented reduced colonic inflammation [13].

In previous work, our group explored the benefits of using 35-kDa molecular weight PEG (PEG35) in experimental models of cold and warm liver ischemia-reperfusion in the rat, finding that the addition of PEG35 to the organ preservation solution decreased liver graft vulnerability to cold ischemia-reperfusion injury [14]. Furthermore, prophylactic intravenous administration of PEG35 to rats reduced the ischemia-reperfusion-induced hepatic injury associated with the preservation of the mitochondrial status, cytoskeleton protection, and the induction of cyto-protective signaling pathways [15]. Intravital microscopy studies demonstrated the location of PEG35 adsorbed in the liver vascular bed after ischemia-reperfusion.

Given the numerous benefits of PEGs just described, and in view of the fact that they are water-soluble and above all non-toxic, the objective of this paper was to study the potentially protective effects of PEG35 in an experimental model of ANP. Our results suggest that PEG35 strongly reduces ANP severity and improves the associated inflammatory process in the lung.

## 2. Results

### 2.1. Effects of PEG35 on Plasma Lipase Levels

**Figure 1:**
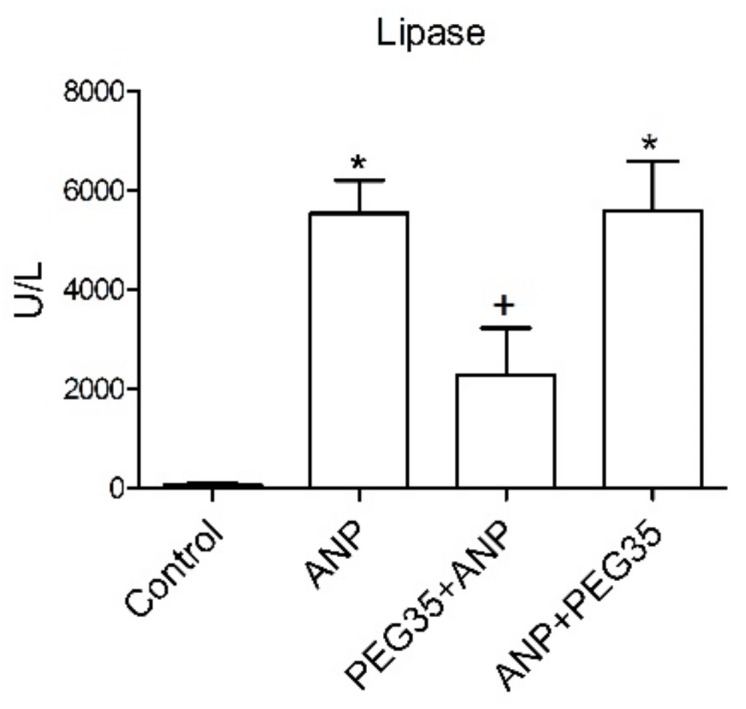
Effect of PEG35 treatment on plasma lipase activity in sodium taurocholate-induced ANP. Plasma lipase levels in U/L. Bars represent mean values of each group ± SEM. * *p* < 0.05 versus Control, + *p* < 0.05 versus ANP. ANP, Acute Necrotizing Pancreatitis. PEG35, Polyethylene glycol 35. Each determination was carried out in triplicate.

Sodium taurocholate-induced ANP in rats was associated with significant increases in the plasma levels of lipase reflecting the degree of pancreatic injury in this experimental model (Figure 1). This increase was significantly reduced in the rats pretreated with 10 mg/kg of PEG35. In contrast, therapeutic PEG35 administration had no effect on the pancreatic injury associated with ANP.

### 2.2. Prophylactic and Therapeutic PEG35 Reduced Systemic Tissue Damage Associated with ANP

**Figure 2:**
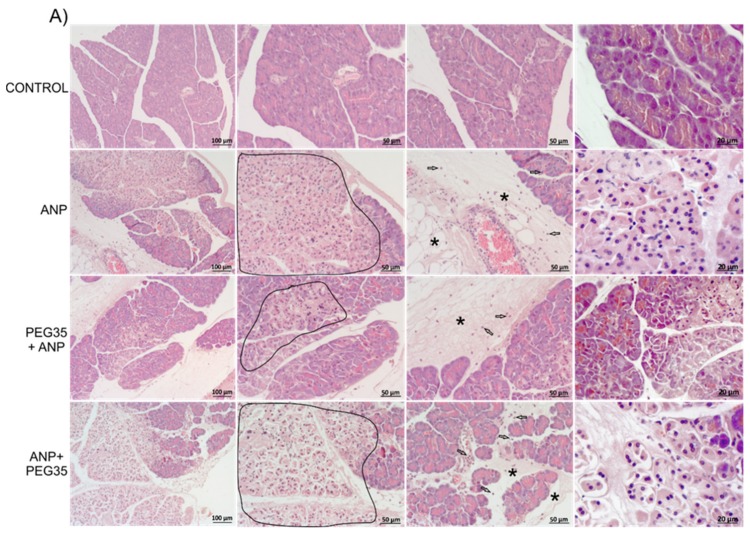

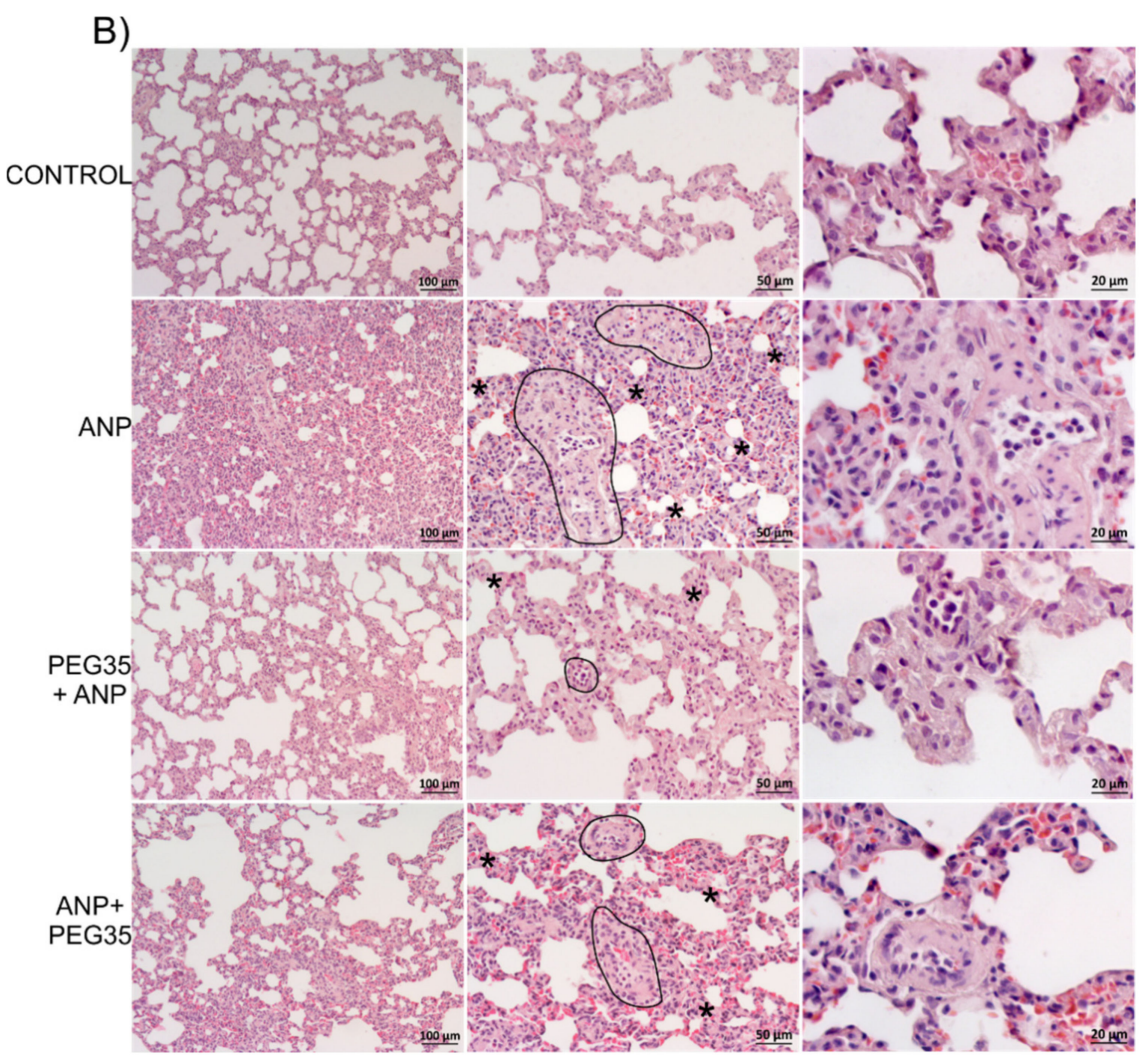
Effect of PEG35 treatment on histological changes in experimental acute necrotizing pancreatitis and associated acute lung injury. (**A**) Representative images of hematoxylin and eosin-stained pancreatic sections for each experimental group. Control group showed normal pancreas structure. ANP and ANP+PEG35 groups presented large areas of necrosis (under area), infiltrated polymorphonuclear neutrophils (indicated by empty arrows) and interstitial edema (indicated by an asterisk). Prophylactic administration of PEG35 significantly reduced these features. (**B**) Representative images of hematoxylin and eosin-stained lung sections for each experimental group. Control group showed normal alveolar structure. In the ANP group, a marked alveolar septal thickening (indicated by an asterisk) with infiltrated neutrophils, and the presence of vessel neutrophils (under area) were seen. Both prophylactic and therapeutic PEG35 treatment normalized alveolar septal thickening and neutrophils infiltration. ANP, Acute Necrotizing Pancreatitis. PEG35, Polyethylene glycol 35. Scale bar, 100, 50 and 20 µM.

Intraductal administration of 5% sodium taurocholate in the rats produced a severe hemorrhagic pancreatitis with large areas of interstitial edema, necrosis and neutrophil infiltration in the pancreas (Figure 2A and Table 1). In the PEG35-treated groups, only when the animals were treated prophylactically were there consistent reductions in pancreatic interstitial edema, leukocyte infiltration and acinar cell necrosis. Histological evaluation of the lungs showed significant edema, leukocyte infiltration and alveolar septal thickening (Figure 2B) associated with ANP. However, these findings were less marked when the animals were treated either prophylactically or therapeutically with PEG35. 

### 2.3. PEG35 Abrogated ANP-Induced Interleukin 6 (IL6) Expression in Plasma

**Figure 3:**
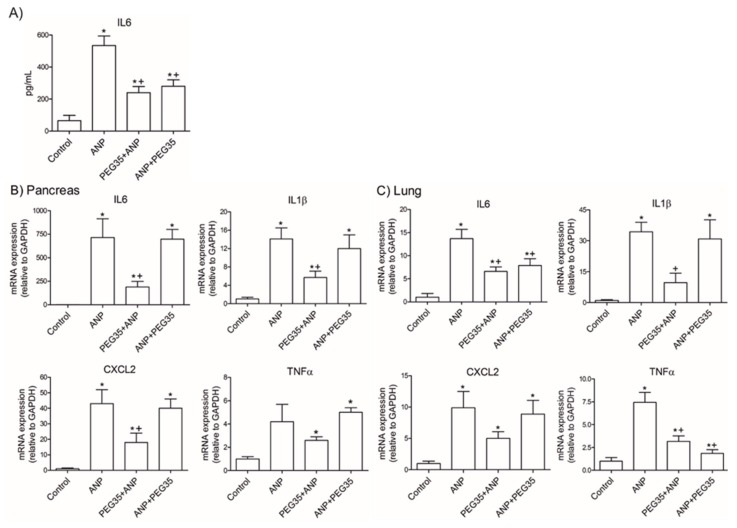
Role of PEG35 on the modulation of pro-inflammatory cytokines and chemokines expression in sodium taurocholate acute necrotizing pancreatitis and associated acute lung injury. (**A**) IL6 expression levels in plasma. (**B**) Pancreatic tissue gene expression of IL6, IL1β, CXCL2 and TNFα by real-time qRT-PCR. (**C**) Lung tissue gene expression of IL6, IL1β, CXCL2 and TNFα by real-time qRT-PCR. In all cases, mRNA induction levels were normalized to GAPDH mRNA expression. Bars represent mean values of each group ± SEM. * *p* < 0.05 versus Control, + *p* < 0.05 versus ANP. ANP, Acute Necrotizing Pancreatitis. PEG35, Polyethylene glycol 35. Each determination was carried out in triplicate.

IL6 is an important multifunctional cytokine with many roles in inflammation, and its serum levels reflect the magnitude of the inflammatory response. This cytokine has been reported to have prognostic value for acute pancreatitis upon admission [16,17]. We therefore measured its levels in plasma and, as expected, a significant increase was detected after ANP induction (Figure 3A). Prophylactic and therapeutic treatment with PEG35 resulted in a significant reduction in systemic IL6 levels.

### 2.4. Prophylactic and Therapeutic PEG35 Improved ANP-Induced Expression of Pro-Inflammatory Cytokines in the Lung

Next, we explored whether PEG35 administration might improve inflammatory response after ANP induction. To do so, we measured the gene expression of pro-inflammatory markers IL6, Interleukin 1β (IL1β), tumor necrosis factor α (TNFα) and chemokine (C-X-C motif) ligand 2 (CXCL2) in both pancreas and lung. Pancreatic tissue levels of these mediators rose markedly three hours after ANP induction compared with control animals (Figure 3B), except for TNFα. As expected, only prophylactic treatment with PEG35 was able to significantly reduce the ANP-induced increases in these cytokines. Regarding the inflammatory process in the lung, ANP induction raised expression levels of IL6, IL1β, TNFα and CXCL2 (Figure 3C). PEG35 administration prior to the induction of ANP significantly reduced IL6, IL1β and TNFα levels, and therapeutic administration significantly reduced the levels of IL6 and TNFα in the lung.

### 2.5. PEG35 Abrogated ANP-Related Adhesion Molecules Expression in the Lung

**Figure 4:**
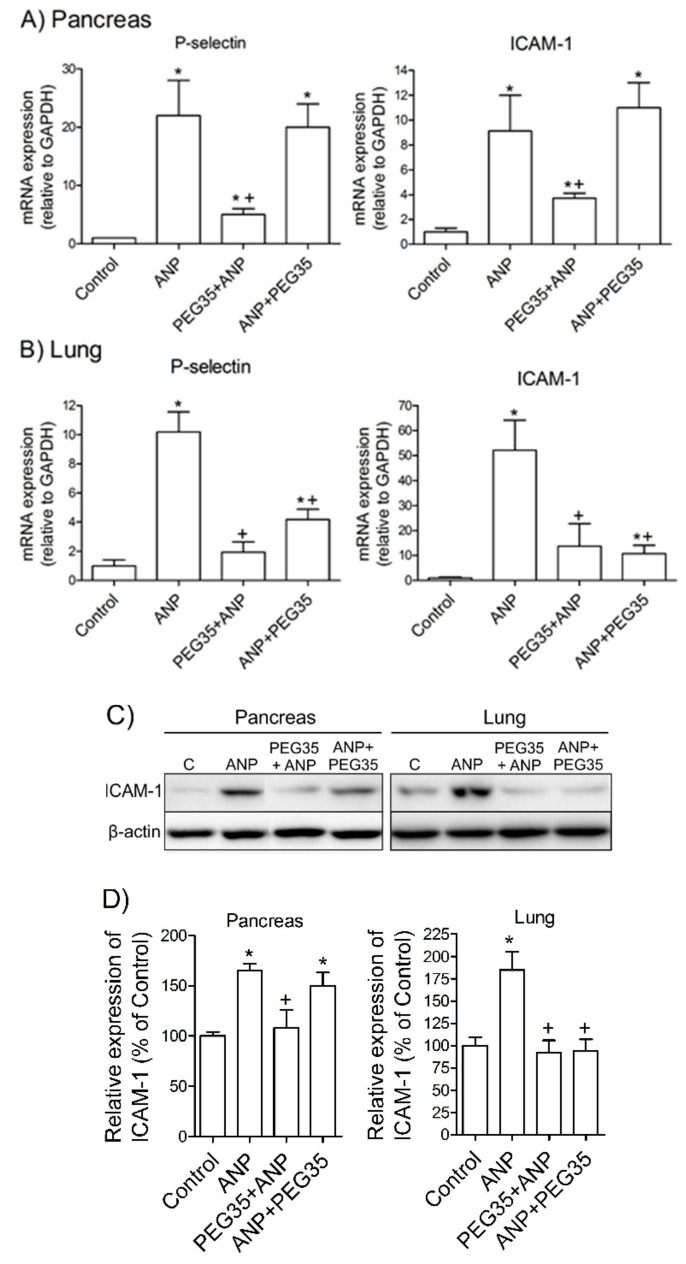
Effects of PEG35 administration on the expression of adhesion molecules in acute necrotizing pancreatitis and associated acute lung injury. (**A**) Pancreatic tissue gene expression of P-selectin and ICAM-1 by real-time qRT-PCR. (**B**) Lung tissue gene expression of P-selectin and ICAM-1 by real-time qRT-PCR. In all cases, mRNA induction levels were normalized to GAPDH mRNA expression. (**C**) Pancreatic and pulmonar protein expression of ICAM-1 assessed by Western Blot analysis. Β-actin expression was used as loading control. Data shown are representative blots for each group. (**D**) Densitometry quantification of Western blot for ICAM-1 in pancreatic and lung tissue. Bars represent mean values of each group ± SEM. * *p* < 0.05 versus Control, + *p* < 0.05 versus ANP. C, Control. ANP, Acute Necrotizing Pancreatitis. PEG35, Polyethylene glycol 35. Each determination was carried out in triplicate.

The recruitment of leukocytes is a hallmark of inflammation. The process is controlled by complex interactions between surface receptors on neutrophils and their corresponding endothelial cell ligands [18]. To further study the protective function of PEG35 in ANP, we focused on the expression of two of the main adhesion molecules involved in this inflammatory disease: P-selectin and Intercellular Adhesion Molecule-1 (ICAM-1) [19]. A significant up-regulation in both adhesion molecules was evident in the pancreas and lung three hours after ANP induction compared to control-operated mice (Figure 4A,B). The prophylactic administration of PEG35 helped to reduce the gene expression of P-selectin and ICAM-1 expression in both these tissues, while its therapeutic administration significantly reduced their expression only in the lung. Accordingly, immunoblot assay of ICAM-1 protein confirmed that PEG35 abrogates the inflammatory process in the lung when is administered either prophylactically or therapeutically (Figure 4C,D).

### 2.6. Prophylactic and Therapeutic Administration of PEG35 Reduced the Pulmonary Neutrophil Infiltration Associated with ANP 

**Figure 5:**
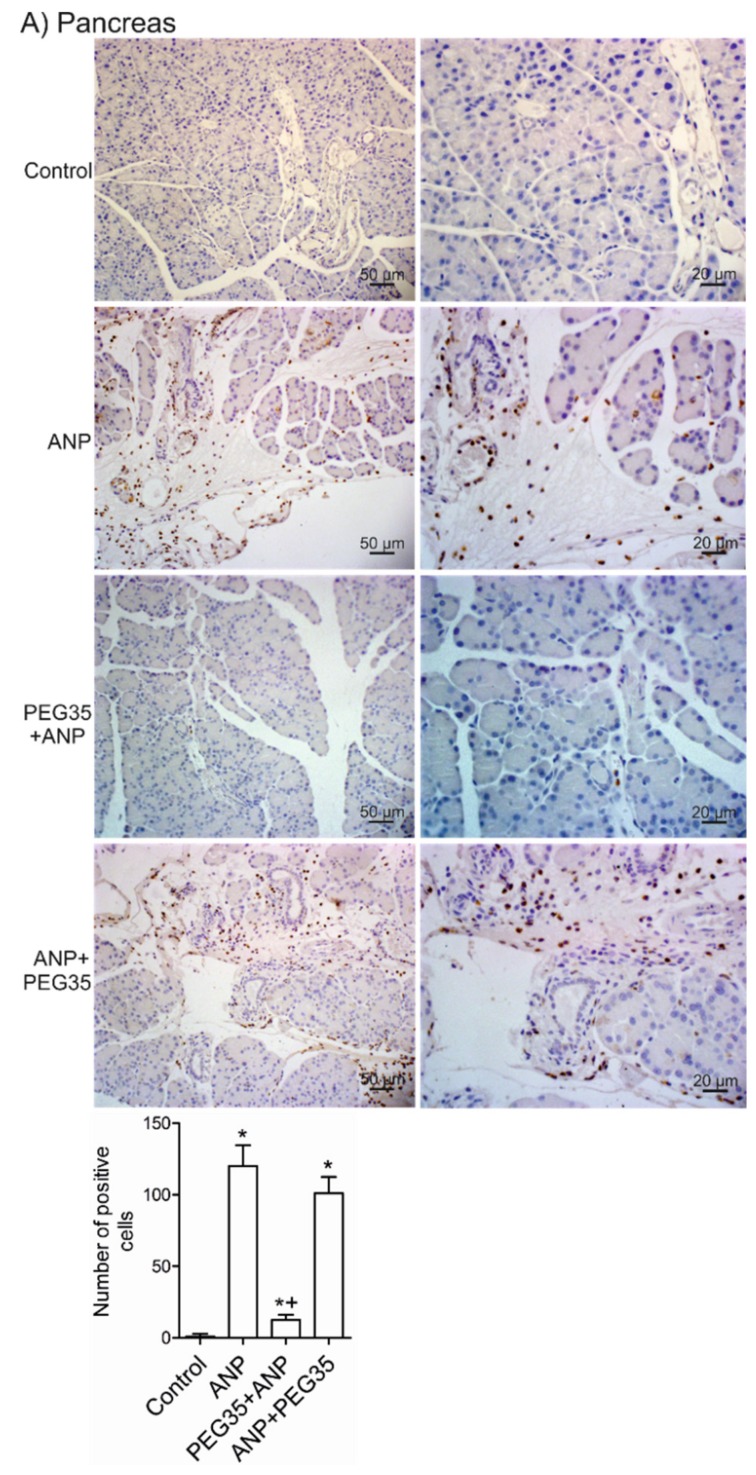

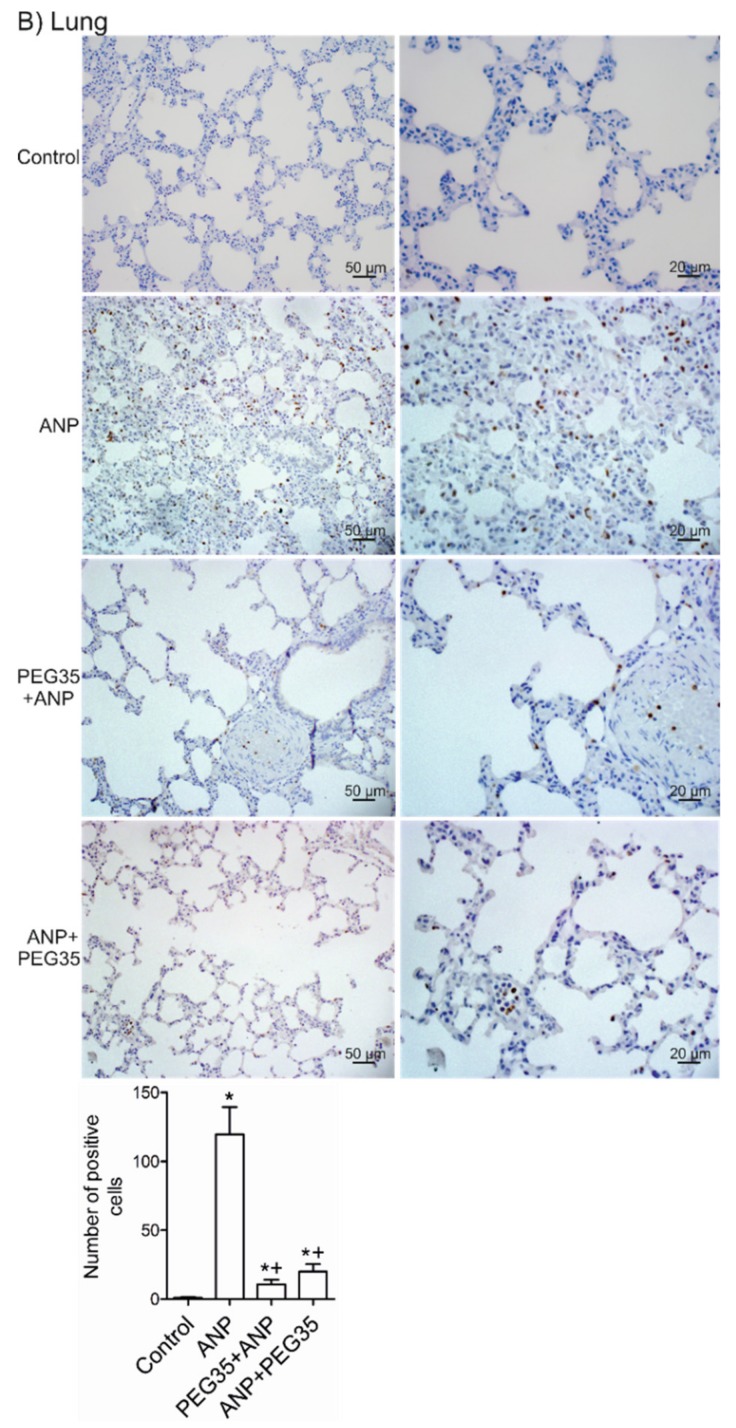
Effects of PEG35 treatment on acute necrotizing pancreatitis-induced myeloperoxidase expression. (**A**) Top, Representative images of pancreatic sections stained with anti-MPO antibody (brown). Bottom, Pancreas MPO immunostaining quantification represented as the average number of positive cells per field. (**B**) Top, Representative images of lung sections stained with anti-MPO antibody (brown). Bottom, Pulmonary MPO immunostaining quantification represented as the average number of positive cells per field. ANP, Acute Necrotizing Pancreatitis. PEG35, Polyethylene glycol 35. Scale bar, 50 and 20 µM. * *p* < 0.05 versus Control, + *p* < 0.05 versus ANP.sss

Increased numbers of neutrophils in both the pancreas (Figure 5A) and lung (Figure 5B) marked the inflammatory response following ANP induction. Additionally, areas of intense cell infiltration with extravasation of leukocytes to the interacinar space were found. Pretreatment with PEG35 significantly attenuated the infiltration of leukocytes into the pancreas. In addition to local pancreatic neutrophil recruitment, a significant increase in myeloperoxidase (MPO) positive cells in the lung was noted in the ANP-induced animals compared to sham-operated mice. By contrast, both prophylactic and therapeutic intravenous administration of PEG35 lessened pulmonary neutrophil recruitment and extravasation.

### 2.7. Effect of PEG35 Treatment on Inflammation-Induced Cell Death

**Figure 6:**
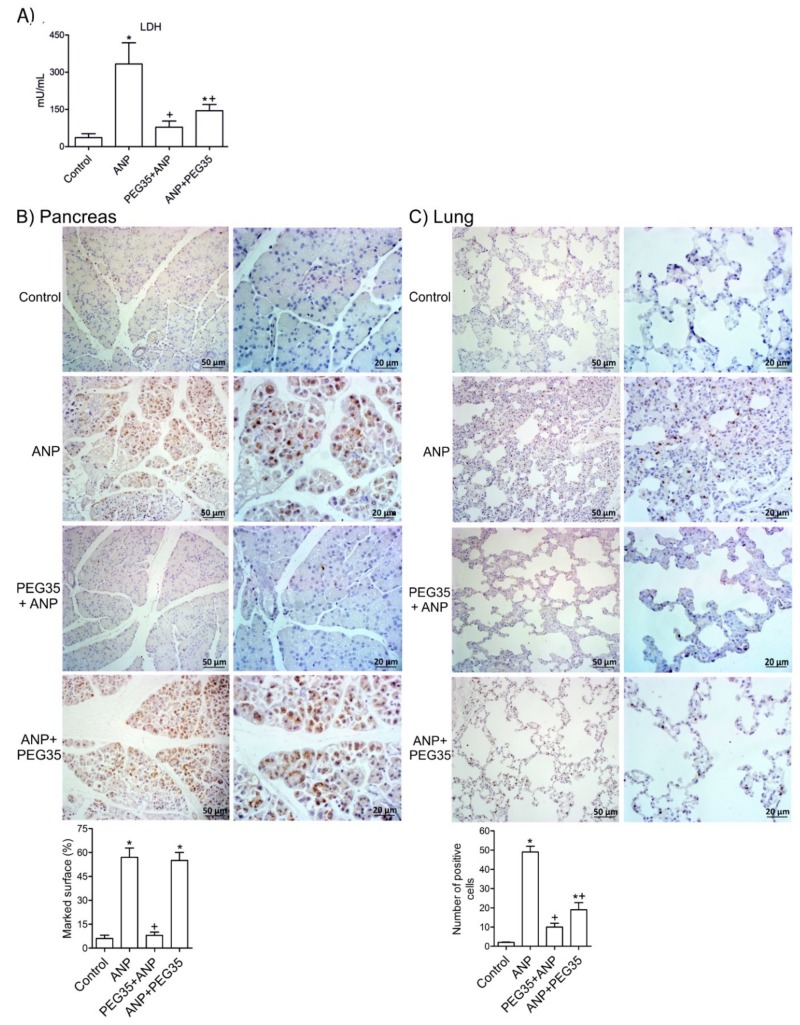
Effects of PEG35 treatment on ANP-induced cell death. (**A**) Plasma lactate dehydrogenase activity three hours after acute necrotizing pancreatitis induction expressed as mU/mL. (**B**) Top: Representative images of pancreatic sections stained with anti-cleaved caspase-3 antibody (brown). Bottom: Pancreas cleaved caspase-3 immunostaining quantification represented as the percentage of marked surface per field. (**C**) Top: Representative images of lung sections stained with anti-cleaved caspase-3 antibody (brown). Bottom: Pulmonary cleaved caspase-3 immunostaining quantification represented as the average number of positive cells per field. Bars represent mean values of each group ± SEM. * *p* < 0.05 versus Control, + *p* < 0.05 versus ANP. ANP, Acute Necrotizing Pancreatitis. PEG35, Polyethylene glycol 35. Each determination was carried out in triplicate.

To further explore the potential protective effects of PEG35 on pancreas and lung, cell necrosis and apoptosis were determined through LDH release and caspase 3 activity respectively. As illustrated in Figure 6A, a significant increase in LDH activity in plasma occurred three hours after ANP induction. Similarly, cleaved caspase-3 levels were markedly higher following ANP induction both in the pancreas and in the lung, compared with the control group (Figure 6B,C). Levels of both necrotic and apoptotic cell markers were significantly reduced under conditions of prophylactic administration with PEG35 in both these tissues. The therapeutic administration of PEG35 significantly lessened LDH levels as well as cleaved caspase-3 expression in the lung. Taken together, these results suggest that PEG35 exerts both anti-necrotic and anti-apoptotic effects, protecting against inflammation-induced cell death following ANP.

## 3. Discussion

Despite extensive research in recent decades, ANP continues to present a significant burden in terms of morbidity, mortality and financial cost, and its management remains a major challenge. Increases in the annual incidence of this disease have been observed in most recent studies and no pharmacological therapies are as yet available to improve the disease course, especially in patients who develop a systemic inflammatory response syndrome. 

Many studies have reported the beneficial effects of PEGs in tissue injury [8,9,11], but the role of these polymers in acute pancreatitis has not yet been elucidated. The present paper aims to establish whether the intravenous administration of a 35-kDa molecular weight PEG in a single non-toxic dose of 10 mg/kg could protect pancreatic and lung tissue against the deleterious effects of ANP. In the study, the prophylactic administration of PEG35 significantly abrogated the severity of acute pancreatitis in sodium taurocholate-treated rats, as indicated by the decreased activity of lipase in plasma. Histopathologic evaluation of the pancreas and systemic lung also revealed a marked reduction in overall histopathology score in the PEG35 pre-treated animals. Surprisingly, the therapeutic administration of PEG35 significantly reduced lung injury, even when the pancreatic lesion was equivalent to that of the untreated ANP-induced group.

Serum levels of pro-inflammatory cytokines and chemokines rise over the course of ANP. IL6 is an important inflammatory mediator of the acute-phase response that has been experimentally associated with distant organ complications [20]. In addition, in the clinical setting, it is considered a reliable early marker for predicting the severity of acute pancreatitis [21,22]. We found that both the prophylactic and therapeutic use of PEG35 was able to significantly abrogate the up-regulated levels of systemic IL6 following ANP induction. Similar results were found regarding the presence of pro-inflammatory cytokines and chemokines locally in the pancreas and in the lung. The gene expressions of IL6, IL1β, and CXCL2 (though not TNFα) were found to be significantly elevated in the pancreas three hours after ANP induction, and prophylactic PEG35 administration abrogated these up-regulated cytokines and chemokine levels. As expected, the therapeutic administration of PEG35 did not have any protective effect on the injured pancreas; in ANP, once the pro-inflammatory cascade is triggered, the process is exceedingly difficult to reverse. With regard to the inflammatory process in the lung, the induction of ANP increased the levels of expression of IL1β, TNFα and CXCL2 compared with the control-operated group. PEG35 administration prior to the induction of ANP significantly reduced IL6, IL1β and TNFα mRNA levels. Interestingly, the therapeutic administration of PEG35 was able to downregulate IL6 and TNFα, the main pro-inflammatory cytokines involved in ANP.

Endothelial P-selectin and ICAM-1 are major adhesion molecules that are highly overexpressed during acute pancreatic inflammation and their blockade has been associated with reductions in pancreatic and lung damage [23,24]. In the present study, levels of P-selectin and ICAM-1 expression rose significantly in the pancreas and in the lung three hours after ANP induction. As expected, the pre-treatment of rats with PEG35 abrogated the increased levels of those adhesion molecules in both tissues. Therapeutic administration of PEG35 was unable to reverse the inflammatory process in the pancreas, but was able to do so in the lung. 

These changes in pro-inflammatory processes brought about by PEG35 administration were further emphasized by the marked reduction in neutrophil recruitment and extravasation both in the pancreas and in the lung when PEG35 was administered previously to ANP. As measured by the presence of MPO, PEG35 pre-treatment reduced the number of MPO positive cells in both tissues while (as in the case of the histological score) therapeutic administration of PEG35 only reduced the levels of MPO positive cells in the lung. In fact, neutrophils were found in high numbers within the lung endothelial vessels even though they did not extravasate into the surrounding tissue. In this regard, PEG35 may exert at least part of its protective function through the endothelial cell coating, which may stop neutrophils in the microcirculation entering the interstitium and infiltrating the lung tissue.

Furthermore, both prophylactic and therapeutic use of PEG35 reduced cell death by lowering plasmatic LDH activity and tissue cleaved-caspase-3 expression both in the pancreas and in the lung. These findings are consistent with those of a previous study, which found that PEGs protected against apoptosis when administered intravenously in an animal model of spinal cord injury [25]. PEG 15–20 also has a potent protective antiapoptotic effect in cardiac myocytes exposed to ischemia-reperfusion injury [9]. Additionally, different molecular weight PEGs have been found to protect renal cells against cold-induced cellular necrosis [26], PEG35 being the most effective. All these findings demonstrate that PEG35 may notably alleviate the severity of ANP and protect against inflammation-induced cell death.

By the time of presentation of ANP, pancreatic necrosis is already non-reversible, so the aim is to minimize the systemic inflammatory response syndrome in order to reduce rates of organ failure, morbidity, and mortality. PEG compounds with different molecular weights have been applied topically, orally, and systemically with notable efficacy in a variety of experimental models. Our data highlight the potential therapeutic use of PEG35 to modulate the progression of ANP towards a lethal outcome. We have demonstrated that prophylactic PEG35 improves the inflammatory response in the lung as a direct consequence of attenuating the initial pancreatic injury. In addition, PEG35 exerts a substantial anti-inflammatory role by directly lowering the lung inflammatory response subsequent to acute pancreatitis when administered therapeutically. This feature is particularly relevant in the clinical setting where new therapeutic treatments are urgently required. 

## 4. Materials and Methods

### 4.1. Experimental Animals

Male Wistar rats weighting 200–250 g were housed in a controlled environment with free access to standard laboratory pelleted formula (A04; Panlab, Barcelona, Spain) and tap water. A period of one week was allowed for animals to acclimatize before any experimentation. All procedures were conducted in accordance with European Union regulatory standards for animal experimentation (Directive 2010/63/EU on the protection of animals used for scientific purposes). The Ethical Committee for Animal Experimentation (CEEA, ethic approval number: 211/18, University of Barcelona, 11/04/2018) approved the animal experiments.

#### Animal Model of ANP

The rats were anesthetized with an intraperitoneal injection of pentobarbital at a dose of 50 mg/kg. After a midline laparotomy, a polyethylene catheter connected to an infusion pump was inserted through the duodenum, via the Ampulla of Vater, and 3–4 mm into the biliopancreatic duct. A bulldog clamp was applied to the proximal biliopancreatic duct (near the liver) to prevent infusion into the liver. The experimental model of ANP was induced in the rats (*n* = 8) by retrograde injection of 5% sodium taurocholate in saline solution at 1mL/Kg/1min for 5 min. using an infusion pump (Harvard Instruments, Edenbridge, UK). Control animals (*n* = 8) received saline solution (NaCl 0.9%). This model represents the reference standard of biliary acute pancreatitis, the most common cause of ANP in humans. As previously reported by our group, the infusion of this bile salt at 5% results in lung injury after 3 h of induction. This lung failure is the main contributing factor to early death in patients with ANP [27]. PEG35 was administered intravenously through the penile vein in a single dose of 10 mg/kg either prophylactically (10 min before ANP induction) or therapeutically (10 min after ANP induction) (*n* = 8 for each group). PEG35 was selected based on the literature review. PEG polymers of high molecular weight (≥4000 Da) have been reported to be suitable as potential therapeutic agents. In addition, our group has wide experience in the study of the protective role of PEG35, which is a high molecular weight PEG currently added to preservation solutions for pancreas, liver and kidney transplantation with optimal results for protecting the tissue from ischemia-reperfusion injury [15]. Buprenorphine (0.05 mg/Kg) was intravenously administered as an analgesic immediately before surgery. Three hours after ANP induction, animals were euthanized, and blood was collected in heparinized syringes from the vena cava. Harvested blood was centrifuged, the plasma removed and stored at −80 °C. Three tissue samples from each animal were taken from the head of the pancreas and from the lung. One portion of each tissue was fixed in 10% phosphate-buffered formalin for histological analysis, another portion was frozen and immediately stored at −80 °C for western blot analysis and the last portion was stored in RNAlater solution for real-time PCR analysis.

### 4.2. Histopathological Examination

Pancreatic and lung tissue were fixed in 10% phosphate-buffered formalin and embedded in paraffin. Sections of 3-μm thickness were mounted on glass slides. Slides were dewaxed and rehydrated before staining with hematoxylin and eosin. Then, a pathologist examined multiple randomly chosen microscopic fields from each experimental group in a blinded manner. Sections of pancreas tissue were scored for the severity of pancreatitis based on edema, leukocyte infiltration and necrosis graded on a semi-quantitative scale from no lesion to intense lesion. Lung injury was assessed histologically using a semi-quantitative scale from no lesion to intense lesion for interstitial and intra-alveolar leukocyte infiltration and alveolar septal thickening. The semi-quantitative scale was the mean of the lesions in each group: −, no lesion; +, slight lesion; +/−, slight lesion in some sections; ++, moderate lesion; +++, intense lesion.

### 4.3. Biochemical Determinations

#### 4.3.1. Lipase Activity

Plasma lipase activity were determined using a commercial turbidimetric assay kit from Randox (County Antrim, Crumlin, UK), according to the supplier’s specifications. Briefly, the degradation of triolein by the pancreatic lipase results in decreased turbidity, which was measured in the sample at 340 nm using an automated microplate reader (iEMS Reader MF; Labsystems, Helsinki, Finland). The activity of the samples was obtained in U/L. All samples were run in duplicate.

#### 4.3.2. IL6 Immunoassay

Interleukin-6 (IL6) in plasma was measured using a commercially available ELISA Kit (R&D Systems, Minneapolis, MN, USA) in accordance with the manufacturer’s instructions. Briefly, standards, control, and samples reacted with a specific antibody against IL6 immobilized in a microplate. Another antibody specific for rat IL6 was then added to the wells. After washing, a substrate solution was added, yielding a yellow product. The intensity of the color measured is in proportion to the amount of IL6. The optical density was measured at 450 nm using an automated microplate reader (iEMS Reader MF; Labsystems, Helsinki, Finland). IL6 levels were obtained in pg/mL. All samples were run in duplicate. 

#### 4.3.3. Lactate Dehydrogenase Activity

Lactate dehydrogenase (LDH) is a soluble cytosolic enzyme present in most eukaryotic cells, and is released upon cell death due to damage to the plasma membrane. LDH activity was measured in samples of plasma using the Lactate Dehydrogenase Assay Kit (Abcam; Cambridge, UK). In this assay, LDH reduces NAD to NADH which then interacts with a specific probe to produce color. Changes in absorbance due to NADH formation were recorded at 450 nm at 37 °C using an automated microplate reader (iEMS Reader MF; Labsystems, Helsinki, Finland). The activity of the samples was expressed in milliunits per milliliter (mU/mL). All samples were run in duplicate. The lower limit of detection for ELISA ranged from 14 to 36 mU/mL.

#### 4.3.4. Real-Time qRT-PCR

Total RNA from the pancreas and lungs was extracted with TRIzol reagent (Invitrogen, Carlsbad, CA, USA) according to the manufacturer’s protocol. RNA concentration and quality were measured with the OD A260/A280 ratio and OD A260/A230 ratio respectively, and the integrity of 18S and 28S ribosomal bands for all RNA preparations was verified by running a 1% agarose gel electrophoresis. Reverse transcription was conducted on a 1 µg RNA sample using the iScript cDNA Synthesis Kit (Bio-Rad Laboratories, Hercules, CA, USA). Subsequent PCR amplification was conducted using SsoAdvanced™ Universal SYBR^®^ Green Supermix (Bio-Rad Laboratories, Hercules, CA, USA) on a CFX Real-Time PCR Detection System (Bio-Rad Laboratories, Hercules, CA, USA) using 10µL of amplification mixtures containing 50 ng of reverse-transcribed RNA and 250 nM of the corresponding forward and reverse primers. PCR primers for the detection of Interleukin 6 (IL6), Interleukin 1β (IL1β) and glyceraldehyde-3-phosphate dehydrogenase (GAPDH) were experimentally validated primers from BioRad (Hercules, CA, USA). PCR primers for Chemokine (C-X-C motif) ligand 2 (CXCL2), Tumor necrosis factor α (TNFα), P-selectin and Intercellular adhesion molecule-1 (ICAM-1) were designed with Primer3.0 plus [28]. The sequences were as follows: CXCL2 forward, 5′-TGCTCAAGACTCCAACCACTC-3′ and reverse 5′- CACAACAACCCCTGTACCCTG-3′; TNFα forward, 5′- ATGGGCTCCCTCTCATCAGT-3′ and reverse 5′-GCTTGGTGGTTTGCTACGAC-3′; P-Selectin forward, 5′-TCTCCTGCAACGAGGAGTTT-3′ and reverse 5′-GGTGTCGACAGGACATTGTG-3′; and ICAM-1 forward, 5′-GAGCGACATTGGGGAAGACA-3′ and reverse 5′- CACTCGCTCTGGGAACGAATA-3′. The specificity of the amplicons was determined by melting curve analysis. Reactions were carried out in duplicate and threshold cycle values were normalized to glyceraldehyde-3-phosphate dehydrogenase (GAPDH) gene expression. The ratio of the relative expression of target genes to GAPDH was calculated by the DCt formula.

#### 4.3.5. Immunohistochemistry

Pancreatic and lung tissue were fixed and embedded in paraffin slices. Sections of 3 μm thickness were then deparaffinized in xylene, rehydrated with graded ethanol, and washed in Tris-buffered saline. After quenching endogenous peroxidase activity and blocking non-specific binding, antigen retrieval was conducted by incubating samples with 10 mM sodium citrate buffer (pH 6.0). Then, the tissue sections were incubated overnight with the myeloperoxidase antibody (1:100 dilution; reference ab9535 Abcam; Cambridge, UK) and the rabbit cleaved caspase-3 (Asp175) antibody (1:800 dilution, reference #9661 Cell signaling, Leiden, The Netherlands). Sections were then incubated with the appropriate dilution of the corresponding biotinylated secondary antibody for 1 h at room temperature. After further washing with Tris-buffered saline, sections were incubated with Vectastain Elite ABC Reagent (Agilent Dako, Inc., Santa Clara, CA, USA) for 30 min at room temperature. Chromogenic immunolocalization was conducted using 0.05% 3,3′-diaminobenzidine (DAB). All sections were counterstained with hematoxylin, dehydrated and mounted. Negative controls were included by replacing the primary antibody with non-immune serum. Images were taken with a Nikon Eclipse E1000 microscope (Nikon, Amsterdam, Netherlands) and analyzed using cellSens imaging software (Olympus, Hamburg, Germany). The mean number of peroxidase-positive cells was counted in six randomly chosen microscopic high-power fields (20×) per animal in a blinded fashion. For pancreatic cleaved caspase-3 analysis, the percentage of the thresholded area occupied by DAB staining was measured using the Image J program.

#### 4.3.6. Western Blot

Pancreas and lung tissue were homogenized in ice-cold RIPA buffer (50 mM Tris-HCl, 150 mM NaCl, 0.05% Triton X-100, 1 mM Ethylenediamine Tetraacetic Acid, 1 mM Dithiothreitol, 1 mM Phenylmethylsulfonyl fluoride, 1 mM NaF, 1mM Na_3_VO_4_, 1 µg/mL Aprotinin, 1 µg/mL Leupeptin; pH 7.4). Lysates were then centrifuged at 15,000× *g* for 20 min at 4 °C, and the supernatants were collected. Protein concentration of the supernatants was determined by the Bradford protein assay (Bio-Rad Laboratories, Hercules, CA, USA). SDS-PAGE was performed on a 10% gel on which 40 µg of total protein per well was loaded. After SDS-PAGE, the proteins were transferred to a polyvinylidene difluoride membrane. Immunoblotting was performed using the mouse monoclonal ICAM-1 antibody conjugated to HRP (dilution 1:100, Santa Cruz Biotechnology, sc-8439 HRP) and β-actin-HRP conjugated (dilution 1:20000, Sigma, A3854). The bound antibody was detected using enhanced chemiluminescence (ECL) detection (Bio-Rad Laboratories, Hercules, CA, USA), and the bands were analyzed using ChemiDoc™ Touch Imaging System (Bio-Rad Laboratories, Hercules, CA, USA). For quantification, protein expression of ICAM-1 was normalized to β-actin.

### 4.4. Statistical Analysis

All data were exported into Graph Pad Prism 4 (GraphPad Software, Inc.) and were presented as means ± SEM. Statistical analyses were carried out by one-way analysis of variance (ANOVA), followed by Tukey’s multiple comparison test to determine the significance between pairs. The minimal level of significance was considered at *p* < 0.05.

## Figures and Tables

**Table 1:** Pancreatic and pulmonary lesions in all experimental groups.

		Pancreas			Lung	
	Edema	Inflammation	Necrosis	Alveolar wall thickening	Infiltrated neutrophils	Vessel neutrophils
Control	+/-	-	-	+/-	+/-	+/-
ANP	+++	+++	+++	+++	+++	+++
PEG35+ANP	+/-	+	+/-	+/-	+/-	+
ANP+PEG35	+++	+++	+++	+	+	+++

Abbreviations: ANP, Acute Necrotizing Pancreatitis. PEG35, Polyethylene glycol 35.

## Abbreviations

ANP	Acute Necrotizing Pancreatitis
PEG35	Polyethylene glycol 35
IL6	Interleukin 6
TNFα	Tumor Necrosis Factor α
IL1β	Interleukin 1β
CXCL2	Chemokine (C-X-C motif) ligand 2
ICAM-1	Intercellular Adhesion Molecule -1
LDH	Lactate Dehydrogenase
MPO	Myeloperoxidase
